# A Model Factory

**Published:** 1901-10-12

**Authors:** 


### A MODEL FACTORY.

To meet the requirements of their business, the Mazawattee Tea Company, Limited, have caused to be erected at New Cross an extensive block of buildings in which they have included a complete and up-to-date department for the manufacture of cocoa and chocolate, and on the occasion of the opening of these factories on the 1st inst., an inaugural uncheon was given, to which many members of the press were invited. Although the factories exist primarily for business purposes, the directors are to be complimented on the fact that cleanliness and healthfulness have not been neglected, for nowhere is there left a loophole for dirt and contamination to enter. All the employees are dressed in neat washing costumes, and everything that could promote healthful working conditions has been realised in these factories. It is not within the scope of this journal to enter into details of the wonderful machinery which has been installed in these works; but if the most up-to-date appliances and the most scrupulous care and regard for cleanliness throughout the whole process of manufacture (it is a rule of the firm that under no circumstances whatever shall the article in course of manufacture be touched by hand) can do anything towards producing a perfect cocoa, then the Mazawattee Lateriba Cocoa should have a great future before it. The cocoa itself we hope to examine and report upon in due course.

